# Rapid methods for extracting and quantifying phenolic compounds in citrus rinds

**DOI:** 10.1002/fsn3.210

**Published:** 2015-10-01

**Authors:** Lembe Samukelo Magwaza, Umezuruike Linus Opara, Paul J. R. Cronje, Sandra Landahl, Jose Ordaz Ortiz, Leon A. Terry

**Affiliations:** ^1^ Department of Crop ScienceSchool of Agricultural, Earth and Environmental SciencesUniversity of KwaZulu‐NatalPrivate Bag X01, Scottsville 3209PietermaritzburgSouth Africa; ^2^ Postharvest Technology Research LaboratoryDepartment of Horticultural ScienceStellenbosch UniversityStellenbosch7602South Africa; ^3^ Postharvest Technology Research LaboratoryDepartment of Food ScienceSouth African Research Chair in Postharvest TechnologyStellenbosch UniversityStellenbosch7602South Africa; ^4^ Citrus Research InternationalDepartment of Horticultural ScienceStellenbosch UniversityStellenbosch7602South Africa; ^5^ Plant Science LaboratoryCranfield UniversityBedfordshireMK43 0ALUK

**Keywords:** Extraction method, flavanone glycosides, phenolic acids, phenolics

## Abstract

Conventional methods for extracting and quantifying phenolic compounds in citrus rinds are time consuming. Rapid methods for extracting and quantifying phenolic compounds were developed by comparing three extraction solvent combinations (80:20 v/v ethanol:H_2_O; 70:29.5:0.5 v/v/v methanol:H_2_O:HCl; and 50:50 v/v dimethyl sulfoxide (DMSO):methanol) for effectiveness. Freeze‐dried, rind powder was extracted in an ultrasonic water bath at 35°C for 10, 20, and 30 min. Phenolic compound quantification was done with a high‐performance liquid chromatography (HPLC) equipped with diode array detector. Extracting with methanol:H_2_O:HCl for 30 min resulted in the optimum yield of targeted phenolic acids. Seven phenolic acids and three flavanone glycosides (FGs) were quantified. The dominant phenolic compound was hesperidin, with concentrations ranging from 7500 to 32,000 *μ*g/g DW. The highest yield of FGs was observed in samples extracted, using DMSO:methanol for 10 min. Compared to other extraction methods, methanol:H_2_O:HCl was efficient in optimum extraction of phenolic acids. The limit of detection and quantification for all analytes were small, ranging from 1.35 to 5.02 and 4.51 to 16.72 *μ*g/g DW, respectively, demonstrating HPLC quantification method sensitivity. The extraction and quantification methods developed in this study are faster and more efficient. Where speed and effectiveness are required, these methods are recommended.

## Introduction

Citrus fruit has a high concentration of natural bioactive compounds with a positive influence on antioxidant capacity (Xu et al. 2008a,b; Tomas‐Barberan and Andres‐Lacueva 2012). As an effective bioactive compound source, rinds of citrus fruit can be explored for health promoting food product values. The phenolic compound profile and concentration in citrus fruit rind has received scientific interest in recent years, due to antioxidant capacity (Manthey and Grohmann 1996; Li et al. 2006; Xu et al. 2008b; Khan et al. 2010; Sun et al. 2010).

The phenolic profile of citrus fruit rinds consists of numerous compounds such as coumarins, psoralens, phenolic acids, and flavonoids (Benavente‐García et al. 1998; Bocco et al. 1998). Flavonoids in citrus rinds are represented by two classes of compounds referred to as flavanone glycosides (FGs) and polymethoxylated flavones (Benavente‐García et al. 1998). These two classes of flavonoids are found only in citrus fruit, and their presence or absence is specific for each species and therefore could be used as taxonomic markers and be related to postharvest physiology (Manthey and Grohmann 1996; Tomás‐Barberán et al. 2003; Mathur et al. 2011). The polymethoxylated flavones occur in relatively lower concentrations but exhibit higher biological activity than phenolic acids and FGs, which are the main primary groups of phenolic compounds in citrus rinds (Benavente‐García et al. 1998; Ma et al. 2008; Simonne and Ritenour 2011; Ye et al. 2011).

An Abundant flavonoid group found in different parts of citrus fruit are FGs including hesperidin, neohesperidin, naringin, narirutin, and didymin (Khan et al. 2010; Jabri‐Karoui and Marzouk 2013). FGs are unique to citrus and are characteristic of some species and varieties (Tomás‐Barberán et al. 2003). A classic example is hesperidin which is a major component in rind tissues of oranges and mandarins. Naringin on the other hand is a predominant FG in grapefruit (Kalt et al. 1999). The concentrations of FGs may differ due to differences in fruit maturity, environmental conditions during growth and development, postharvest treatments, and storage conditions (Abad‐García et al. 2012). Thus, these compounds have a potential to be used as biochemical indicators of fruit origin, species and cultivar.

The health‐related beneficial characteristics of some phenolic compounds have led to a number of studies to develop better extraction, identification, and quantification methods. Many analytical methods are widely used to determine and quantify phenolic compounds in citrus fruit (Ahmad et al. 2006). High‐performance liquid chromatography (HPLC) is the most used technique for analysis of individual compounds (Li et al. 2006).

Extraction of compounds from plant materials is one of the most important steps prior to their determination by HPLC. Conventional extractions are usually time consuming and require relatively large quantities of solvents. It is also well known that the complexity of phenolic compounds in plant matrixes makes extraction difficult (Manthey and Grohmann 1996). In recent years, some novel extraction methods of phenolic compounds have been developed including enzyme‐assisted extraction methods (Li et al. 2006), ultrasound‐assisted extraction (Khan et al. 2010), ultrasonic extraction (Ma et al. 2008), microwave‐assisted extraction (Ahmad et al. 2006), and the use of solvents like dimethyl sulfoxide (DMSO), methanol‐DMSO mixtures, and dimethylformamide (Manthey and Grohmann 1996).

A major trend in modern HPLC is the reduction in particle size and column length to allow very fast separations with greater resolution (Gritti and Guiochon 2012). The use of smaller particles in packed‐column LC to provide increased efficiencies is currently the most prevalent method employed in liquid phase separation (de Villiers et al. 2006). As a result of this new leap forward in column technology, manufacturers began to produce and commercialize shorter columns, down to between 50 and 150 mm, which are as or more efficient than longer columns (Omamogho et al. 2011). This study was therefore conducted to develop a fast faster extraction method and rapid HPLC method for the quantification of phenolic compounds in ‘Nules Clementine’ mandarin rind tissues.

## Materials and Methods

### Chemicals

All chemicals were of analytical grade. Polyphenols (*ρ*‐hydroxybenzoic acid, chlorogenic acid, vanillic acid, caffeic acid, *ρ*‐coumaric acid, ferulic acid, sinapic acid, naringin, and hesperidin) standards were purchased from Sigma Aldrich (Dorset, UK). Narirutin and didymin standards were purchased from Extrasynthese (Lyon, France). Acetonitrile, methanol, and formic acid were all of HPLC grade, dimethyl sulfoxide (DMSO) was analytical grade, and purchased from Fisher Scientific Chemicals (Leics., UK). Solutions and solvents were prepared with Milli‐Q water (Milipore Inc. (Molsheim, France); *σ *= 18 mol/L Ω/cm).

### Plant material and sample preparation

A total of 20 “Nules Clementine” mandarin (*Citrus reticulata* Blanco) fruit were harvested in 2012 from an orchard at Stellenbosch University experimental farm, Western Cape Province, South Africa (33°53′04.56″S, 18°37′36.84″E). These fruit were selected, weighed, peeled, and the rind snap‐frozen in liquid nitrogen and stored at ultra‐low temperature of −80°C. Fresh frozen samples were then freeze‐dried in a Labogene ScanVac CoolSafe Freeze Dryer System (CS55‐4, Lynge, Denmark) for 7 days at 0.015 kPA and −55°C. Lyophilized samples were ground using a pestle and mortar into fine powder. To achieve standard particle size, the ground material was sieved through a 1‐mm metal sieve. Large particles remaining on the sieve were further ground until all the material passed through the sieve. Ground samples were returned into the freezer until extraction and further analysis.

### Polyphenol extraction method

Three different extraction solvent combinations and three extraction times were compared for effectiveness. The extraction solvents included aqueous ethanol [80:20; v/v, ethanol:H_2_O] (Xu et al. 2008a), acidic aqueous methanol [70:29.5:0.5; v/v/v, methanol:H_2_O:HCl] (Crespo et al. 2010), and 50:50; v/v dimethyl sulfoxide (DMSO) as described elsewhere (Manthey and Grohmann 1996; Xu et al. 2008b). Freeze‐dried citrus rind powder (150 ± 0.5 mg) was added into 5 mL solvent following the optimum solvent to solid ratio of citrus fruit prescribed elsewhere (Sun et al. 2010) and put into an ultrasonic water bath (Ma et al. 2008) at 35°C for 10, 20, or 30 min. Samples were agitated for 30 sec every 5 min, centrifuged at 16,000 g force for 10 min before the flocculate was filtered through a 0.2 *μ*m syringe‐driven filter (Millipore corporation, Billerica, MA).

### Extraction recovery and preparation of standard solution

The recovery of different phenolic compounds was evaluated using a pooled rind sample extracted as above. Briefly, freeze‐dried samples were prepared, spiked with specific concentration of naringin and cinnamic acid (16 *μ*g/mL) and extracted in triplicates. The recoveries were calculated based on a method described elsewhere (Chang et al. 1997). The recovery of these phenolic compounds ranged from 94.3% to 103.7%. A mixed standard solution (5 mg/mL) was prepared by transferring all measured phenolic compounds into the extraction solvent. Eight concentration levels of the mixed standard solution were prepared by serial dilution of the stock solution. Concentrations of phenolic acids were determined from linear standard calibration curves (*R* ^2^ = 0.99).

### HPLC quantification of polyphenols

Quantification of phenolic compounds was executed in triplicate on an Agilent 1200 series HPLC equipped with an Agilent DA G1315B/G1365G diode array detector. (DAD) with multiple wavelength detector, degasser and cooled autosampler (Agilent Technologies, Berks, UK). The system was operated by Windows NT‐based ChemStation© software (Agilent Technologies), which was also used for data processing. Citrus rind extracts (20 *μ*L) were injected into a Poroshell 120 column (4.6 × 150 mm and 2.7 *μ*m particle size, Agilent), which was held at 40°C. The flow rate of the mobile phase was set at 1 mL/min. The mobile phases consisted of two solvents, 0.1% (v/v) formic acid: water (A) and 80% (v/v) acetonitrile:water (B). The DAD UV detection of all phenolic acids and FGs was carried out at 280 nm. The solvent gradient conditions for phenolic acids in volume ratios were as follows: 0–5% B during 5 min, 5–10% B up to 10 min; 10–12% B up to 16 min, 12–15% up to 25 min, 15–100% B up to 27 min. For FGs, the solvent gradient conditions were 0–15% during 5 min, 15–20% up to 10 min, 20–60% up to 25 min, and 60–100% up to 27 min. FGs were quantified using naringin (an FG not present in “Nules Clementine” mandarin) as an internal standard. The identification of phenolic compounds was accomplished by comparing the retention times and HPLC spectra of each compound of the peaks in the sample to those of the phenolic compound standards.

### Limit of detection and limit of quantification

The limit of detection (LOD) and limit of quantification (LOQ) for phenolic compounds were calculated by repeatedly (*n* = 10) injecting known concentration of a mixture of standard solution. The LOD and LOQ values were calculated as the amount of each individual phenolic compound required to give the signal to noise ratio of 3:1 and 10:1, respectively (Bressolle et al. 1996).

### Statistical analysis

Statistical analyses were carried out using SPSS 10.0 for Windows (SPSS Inc. Chicago, IL). Data were subjected to analysis of variance (ANOVA). Duncan's multiple‐range tests were used to compare the significant differences in the mean values (*P *≤* *0.05).

## Results and Discussion

### Development of polyphenols extraction method

Dry powder samples of mandarin rind were extracted with 80:20 (v/v) aqueous ethanol compared to acidic aqueous methanol 70:29.5:0.5 (v/v/v; methanol:H_2_O:HCl) and 50:50 (v/v; DMSO:methanol) to determine the efficacy of the extraction procedure for optimum phenolic acid and flavanones yield. Extraction solvent and extraction time were the two main parameters that affected the yield of phenolic compounds (Table 1). The concentration of phenolic acids increased with an increase in ultrasonic extraction time, while flavanones stayed the same. Results showed that an extraction period of 30 min using 70:29.5:0.5 (v/v/v; methanol:H_2_O:HCl) was sufficient to extract phenolic acids. For example, the concentration of ferulic acid after extraction using acidic methanol for 10, 20, and 30 min, gradually increased (12.43, 13.37, 25.19 *μ*/g DM), respectively. The same trend was observed for sinapic acid, where the corresponding concentrations were 41.35, 61.23, and 64.87 *μ*/g DM. In general, phenolic acids yield was higher in samples extracted for 30 min using aqueous methanol. For flavanones, the highest yield was observed in samples extracted using 50:50 (v/v; DMSO:methanol) for 10 min. However, phenolic acids yield was lower using this extraction combination. The concentrations of phenolic acids are similar to those reported by Xu et al. (2008a,b). Therefore, acidic aqueous methanol extraction in ultrasonic bath for 30 min is suitable to extract phenolic acids and 50:50 (v/v; DMSO:methanol) for 10 min was ideal to extract flavanones. By using these methods, extraction time was reduced significantly from 1, 3, 24, and 72 h reported by Xu et al. (2008a), Li et al. (2006), Manthey and Grohmann (1996), and Mathur et al. (2011), respectively.

**Table 1:** Composition of phenolic compounds in rind extracts using different extraction solvents and time combination. Means with different letters in the three rows (solvent) and three columns (extraction times) corresponding to the same compound are significantly different (*P* < 0.05)

Phenolic compound	Extraction solvent	Concentration (*μ*g/g DW)		
		10 min	20 min	30 min
Hydroxybenzoic acids				
*ρ*‐Hydroxybenzoic acid	Methanol	22.08 ± 0.6^ab^ *	19.78 ± 0.7^a^	21.02 ± 3.1^ab^
	DMSO	92.85 ± 1.5^e^	87.75 ± 5.6^d^	86.66 ± 1.9^d^
	Ethanol	29.26 ± 3.5^c^	25.32 ± 1.5^bc^	29.29 ± 0.3^c^
Vanillic acid	Methanol	17.82 ± 0.2^c^	12.69 ± 0.6^b^	24.47 ± 2.7^d^
	DMSO	nd	nd	nd
	Ethanol	8.86 ± 0.8^a^	7.11 ± 1.1^a^	17.25 ± 1.9^c^
Hydroxycinnamic acids				
Chlorogenic acid	Methanol	15.37 ± 0.4^c^	25.91 ± 0.5^e^	43.25 ± 1.2^h^
	DMSO	5.98 ± 1.1^a^	11.89 ± 0.3^b^	33.89 ± 4.4^g^
	Ethanol	18.76 ± 0.9^d^	11.06 ± 0.7^b^	29.85 ± 0.4^f^
Caffeic acid	Methanol	28.21 ± 0.7^e^	23.57 ± 0.5^d^	39.81 ± 3.9^f^
	DMSO	11.95 ± 0.9^a^	12.44 ± 0.2^a^	23.33 ± 1.9^d^
	Ethanol	15.49 ± 1.7^b^	11.94 ± 0.6^a^	19.70 ± 0.8^c^
*ρ*‐Coumaric acid	Methanol	9.63 ± 0.1^c^	14.55 ± 1.8^e^	6.94 ± 0.1^b^
	DMSO	5.63 ± 0.4^ab^	5.35 ± 1.1^a^	9.80 ± 1.3^c^
	Ethanol	4.49 ± 0.2^a^	10.43 ± 0.1^c^	12.53 ± 0.2^d^
Ferulic acid	Methanol	12.43 ± 0.8^b^	13.37 ± 0.8^b^	25.19 ± 4.9^d^
	DMSO	7.92 ± 1.2^a^	6.28 ± 2.5^a^	13.50 ± 1.1^b^
	Ethanol	17.81 ± 0.3^c^	15.81 ± 0.1^bc^	40.55 ± 0.5^e^
Sinapic acid	Methanol	41.35 ± 0.5^e^	61.23 ± 3.8^d^	64.87 ± 2.8^e^
	DMSO	15.19 ± 1.6^a^	23.52 ± 2.4^c^	23.58 ± 2.8^c^
	Ethanol	19.45 ± 4.3^b^	24.99 ± 0.8^c^	39.45 ± 1.1^d^
Flavanones				
Narirutin	Methanol	737 ± 1.4^b^	738 ± 7.9^b^	690 ± 14.4^b^
	DMSO	1370 ± 29.6^d^	1299 ± 140^d^	1151 ± 23.1^c^
	Ethanol	396 ± 30.7^a^	355 ± 11.2^a^	408 ± 12.6^a^
Hesperidin	Methanol	8005 ± 529^cd^	8628 ± 269^d^	7553 ± 290^c^
	DMSO	32,008 ± 373^e^	31,179 ± 1181^e^	32,019 ± 866^e^
	Ethanol	5456 ± 389^b^	4329 ± 439^a^	3966 ± 161^a^
Didymin	Methanol	268 ± 4.6^d^	246 ± 23.6^bc^	257 ± 11.1^cd^
	DMSO	402 ± 7.2^e^	402 ± 9.5^e^	404 ± 4.5^e^
	Ethanol	238 ± 5.1^ab^	224 ± 7.4^a^	232 ± 3.2^ab^

nd, non detectable; *Mean ± SD of three samples.

### Development of HPLC quantification for polyphenols

**Figure 1:**
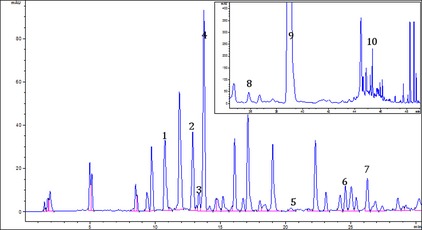
Typical HPLC‐DAD chromatogram at 280 nm showing separation of phenolic compounds in the rind sample (1, *ρ*‐Hydroxybezoic acid; 2, Vanillic acid; 3, Chlorogenic acid; 4, Caffeic acid; 5, *ρ*‐Coumaric acid; 6, Ferulic acid; 7, Sinapic acid; 8, Narirutin; 9, Hesperidin, and 10, Didymin, respectively.

A typical chromatogram with phenolic compounds separation obtained using conditions described earlier is portrayed in Figure 1. A total of seven phenolic acids, including three hydroxybenzoic acids (*ρ*‐hydroxybezoic and vanillic), and five hydroxycinnamic acids (chlorogenic, caffeic, *ρ*‐coumaric, ferulic, and sinapic) as well as three flavanones (narirutin, hesperidin and didymin) were identified and quantified. The method separated 10 phenolic compounds faster (50 min) than 120 min previously reported (Li et al. 2006; Kelebek 2010; Kelebek and Selli 2011). Hesperidin was the dominant compound ranging from 31,179 to 32,019 *μ*g/g DM in samples extracted using DMSO (Table 1). These results are similar to those previously observed by Xu et al. (2008a,b), who reported a total of seven phenolic acids and four flavanones. The flavanones profile was similar to that reported by Ye et al. (2011) who reported hesperidin as the major flavanone in mandarin fruit.

Table 2 summarizes the concentration range, retention times, regression equation (*y* = *mx*), coefficient of determination (*R* ^2^), LOD, LOQ, and the relative standard deviation (RSD) for each compound. The reproducibility of the retention time of phenolic compounds under selected HPLC conditions was executed by doing repeated injections (*n* = 10) of the mixture of the 10 standards at the concentration of 10.0 *μ*g/mL. The regression equation, LOD, LOQ, and RSD were calculated for each identified phenolic compound using only the best extraction method, which in this case was acidic methanol. The LOD, defined as the smallest concentration that the analytical procedure can reliably distinguish from the noise levels and LOQ for all analytes were very small, ranging from 1.35 to 5.02 and 4.51–16.72 *μ*g/mL, respectively. The RSD values for all retention times ranged from 0.45 to 1.67 indicating good stability and adequate performance of the method investigated.

**Table 2:** Response characteristics of phenolic compound standards using HPLC. In the regression equation, *x* represents concentration of phenolic compounds and *y* represents the peak area. The linear standard concentration range was between 5 and 150 *μ*g/mL (5, 16, 20, 60, 100, 150). The presented values LOQ, LOD, and RSD were measured with repeated injections (*n* = 10) of standard mixture at a concentration of 10 *μ*g/mL each

Phenolic compound	Retention time	Regression equation	*R*²	LOD (*μ*g/mL)	LOQ (*μ*g/mL)	R.S.D (%)
Hydroxybenzoic acids						
*ρ*‐Hydroxybenzoic acid	11.5	*y* = 23.45*x*	0.9997	1.48	4.92	0.49
Vanillic acid	14.1	*y* = 30.62*x*	0.9995	1.39	4.62	0.46
Hydroxycinnamic acids						
Chlorogenic acid	14.4	*y* = 33.83*x*	0.9997	1.48	4.92	0.49
Caffeic acid	14.9	*y* = 62.73*x*	0.9995	1.35	4.51	0.45
*ρ*‐Coumaric acid	20.2	*y* = 96.81*x*	0.9997	1.45	4.84	0.48
Ferulic acid	25.0	*y* = 60.076*x*	0.9997	1.42	4.74	0.47
Sinapic acid	26.9	*y* = 27.45*x*	0.9990	2.32	7.73	0.77
Flavanones^a^						
Narirutin	17.0	*y* = 30.83*x*	0.9994	1.50	3.01	0.50
Didymin	24.8	*y* = 31.13*x*	0.9994	1.32	4.23	0.45
Hesperidin	21.0	*y* = 30.84*x*	0.9994	5.02	16.72	1.67

a Flavanones were determined on a different HPLC run.

## Conclusions

Rapid and efficient methods for extracting and quantifying phenolic compounds in citrus rinds were successfully developed. Aqueous acidic methanol and 50:50 (v/v; DMSO:methanol, respectively) extract phenolic acids and flavanone glycosides rapidly and efficiently. The HPLC method developed in this study separated faster than methods previously described. Phenolic compounds can be extracted rapidly and efficiently from citrus rind tissue.

## Conflict of Interest

None declared.
